# Association Between Post-procedure Cerebral Blood Flow Velocity and Severity of Brain Edema in Acute Ischemic Stroke With Early Endovascular Therapy

**DOI:** 10.3389/fneur.2022.906377

**Published:** 2022-07-18

**Authors:** Jie Pan, Huadong Wu, Tingting Wu, Yu Geng, Ruozhen Yuan

**Affiliations:** ^1^Suzhou Medical College of Soochow University, Suzhou, China; ^2^Center for Rehabilitation Medicine, Department of Neurology, Zhejiang Provincial People's Hospital (Affiliated People's Hospital, Hangzhou Medical College), Hangzhou, China

**Keywords:** ischemic stroke, endovascular therapy, edema, cerebral blood flow velocity, transcranial Doppler

## Abstract

**Objectives:**

We aimed to investigate the association between post-procedure cerebral blood flow velocity (CBFV) and severity of brain edema in patients with acute ischemic stroke (AIS) who received early endovascular therapy (EVT).

**Methods:**

We retrospectively included patients with AIS who received EVT within 24 h of onset between February 2016 and November 2021. Post-procedure CBFV of the middle cerebral artery was measured in the affected and the contralateral hemispheres using transcranial Doppler ultrasound. The severity of brain edema was measured using the three-level cerebral edema grading from the Safe Implementation of Thrombolysis in Stroke-Monitoring Study, with grades 2–3 indicating severe brain edema. The Association between CBFV parameters and severity of brain edema was analyzed.

**Results:**

A total of 101 patients (mean age 64.2 years, 65.3% male) were included, of whom 56.3% (57/101) suffered brain edema [grade 1, 23 (22.8%); grade 2, 10 (9.9%); and grade 3, 24 (23.8%)]. Compared to patients with non-severe brain edema, patients with severe brain edema had lower affected/contralateral ratios of systolic CBFV (median 1 vs. 1.2, *P* = 0.020) and mean CBFV (median 0.9 vs. 1.3, *P* = 0.029). Multivariate logistic regression showed that severe brain edema was independently associated with affected/contralateral ratios of systolic CBFV [odds ratio (OR) = 0.289, 95% confidence interval (CI): 0.069–0.861, *P* = 0.028] and mean CBFV (OR = 0.278, 95% CI: 0.084–0.914, *P* = 0.035) after adjusting for potential confounders.

**Conclusion:**

Post-procedure affected/contralateral ratio of CBFV may be a promising predictor of brain edema severity in patients with AIS who received early EVT.

## Introduction

Brain edema is one of the most devastating complications after acute ischemic stroke (AIS) (1). Malignant brain edema (MBE) is the most severe type of brain edema, and it usually occurs following the occlusion of the internal carotid artery (ICA) or the middle cerebral artery (MCA) (2). MBE is characterized by a disastrous clinical course, with a mortality rate reaching 80% in conservatively treated patients (3). Since effective treatment options are limited once MBE occurs, early prediction and identification of patients at risk of severe brain edema is essential to improve prognosis (4).

Endovascular therapy (EVT) is the most effective treatment for patients with large vessel occlusion (5). Recent studies showed that revascularization was associated with a reduced risk of MBE (4) and that EVT was inversely associated with the use of decompressive craniectomy for patients with AIS (6). However, about 20% of patients with large vessel occlusion still developed MBE despite successful recanalization (7, 8). Whether post-procedure factors could guide the early prediction of brain edema severity is still unknown for patients receiving EVT.

Transcranial Doppler (TCD) as a non-invasive approach for measuring cerebral blood flow velocity (CBFV) and estimating increased intracranial pressure at the bedside is widely used in neurocritical care (9, 10). CBFV reflects real-time cerebral hemodynamics in patients with AIS; however, the value of CBFV in predicting brain edema has been poorly studied (11). Therefore, in this study, we aimed to investigate the value of post-procedure CBFV in predicting the severity of brain edema in patients with AIS who received early EVT.

## Materials and Methods

### Patient Selection

Patients with AIS who were admitted to Zhejiang Provincial People's Hospital (Hangzhou, China) from February 2016 to November 2021 and received early EVT were prospectively included in our study. Stroke was diagnosed according to the World Health Organization criteria, and ischemic stroke was confirmed by brain computed tomography (CT) or magnetic resonance imaging (MRI). We included patients who received EVT for occlusion of the MCA or ICA within 24 h of stroke onset. We excluded patients who had more than one EVT within 1 week of stroke onset, who had no imaging scan or TCD examination within 1 week of EVT, or who underwent post-procedure TCD after the last CT scan.

This study was approved by the Ethics Committee of Zhejiang Provincial People's Hospital. Informed consent was obtained from patients or their relatives.

### Clinical Management and Data Collection

Upon admission, a brain non-contrast CT scan was performed for every patient with AIS. Patients who were suspected of large vessel occlusion were further evaluated by CT angiography and CT perfusion scanning (Aquilion/ONE TSX-301A, Toshiba, Tokyo, Japan). We conformed to the latest guidelines for the selection of candidates for early EVT (5, 12). The final decision to perform EVT was made after discussing it with the patient's families. In our center, EVT includes mechanical thrombectomy with a stent retriever, direct aspiration, and angioplasty/stenting. Which procedure to perform is determined by the neuro-interventionalist in charge.

From all patients, we collected demographic information, time of onset, past medical history, and stroke severity on admission. Stroke severity was measured using the National Institutes of Health Stroke Scale (NIHSS) (13) and Glasgow Coma Scale (GCS) (14). The state of post-procedure recanalization was evaluated using the modified Treatment in Cerebral Ischemia (mTICI) score (15). Hemorrhagic transformation was determined using the European Cooperative Acute Stroke Study (ECASS) criteria (16), which comprised hemorrhagic infarction and parenchymal hemorrhage.

Post-procedure CBFV of the MCA was measured using TCD (EMS-9PB, Delica, Shenzhen, China) within seven days of stroke onset. We used a 2-MHz probe to obtain the CBFV of bilateral MCAs through the temporal bone window, with a depth of 45–60 mm (9). Systolic, diastolic, and mean CBFVs were recorded. We performed a brain CT scan immediately after EVT. We strictly maintained post-procedure blood pressure under 140/90 mmHg in all patients receiving EVT. If angioplasty or stenting was performed during EVT, blood pressure was further controlled around 110–120/70–80 mmHg. If the patient met indications for decompressive craniectomy, neurosurgeons were consulted.

Osmotic therapy was prescribed when the radiological sign of space-occupying brain edema was observed. Mannitol and/or glycerol infusion was the most used regimen. The dosage of osmotic therapy was determined by the neurologist in charge. Follow-up brain CT/MRI was scheduled on the first, third, and seventh days after EVT, or in case of neurological worsening.

### Outcome Measures

**Figure 1:**
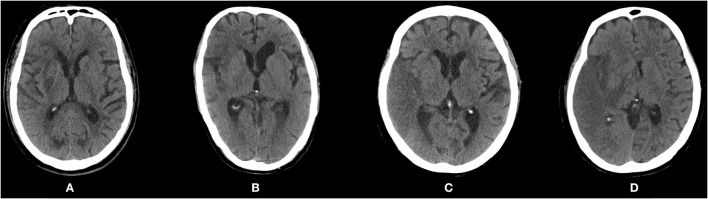
Post-procedure computed tomography scans showing different severity of brain edema according to the Safe Implementation of Thrombolysis in Stroke-Monitoring Study (SITS-MOST). **(A)** CED-0: no edema. **(B)** CED-1: focal brain edema up to one-third of the hemisphere. **(C)** CED-2: brain edema greater than one-third of the hemisphere. **(D)** CED-3: brain edema with midline shift. CED, cerebral edema.

Brain edema was defined as effacement of cortical sulci or the ventricular system due to compression of adjacent brain tissue (17). The severity of brain edema was further assessed using the cerebral edema (CED) grading from the Safe Implementation of Thrombolysis in Stroke-Monitoring Study (SITS-MOST) (18), a three-level scale that classifies brain edema into CED-1 (focal brain edema up to one-third of the hemisphere), CED-2 (brain edema greater than one-third of the hemisphere), and CED-3 (brain edema with midline shift, MLS). Typical imaging observations of different severity grades of brain edema are shown in Figure 1.

The primary outcome of this study was severe brain edema (SBE), defined as brain edema of grades CED-2 or CED-3. The secondary outcome was MBE, defined as clinical deterioration (decrease in NIHSS ≥ 4 points or decrease in NIHSS item 1a of ≥1 point), together with radiological signs of space-occupying edema (MLS ≥ 5 mm) within seven days of onset (4). MLS was defined as the distance of septum pellucidum displacement at the level of the Foramen of Monro (19).

### Statistical Analyses

All statistical analyses were performed using SPSS version 23.0 (IBM, Armonk, NY, USA). We reported the mean ± standard deviation (SD) or median with interquartile range (IQR) for reporting continuous variables, and number with percentage for categorical variables. All ratios presented throughout the article indicate the proportion of the parameter in the affected hemisphere compared with the contralateral hemisphere.

Baseline variables were compared between groups with different severity of brain edema (SBE vs. non-SBE, MBE vs. non-MBE). Analysis of variance (ANOVA) or Mann-Whitney *U* test were used to compare continuous variables, and χ^2^ or Fisher's exact test were used to compare categorical variables as appropriate. Multivariate logistic regression was applied to examine the association between CBFV and severity of brain edema after adjustment for potential confounders. Multivariate analysis was conducted using variables that were associated with *P* < 0.10 in the univariate analysis, as well as variables previously linked to brain edema (4, 20, 21). Consequently, we selected age, NIHSS score, and atrial fibrillation as potential confounders. Correlations between TCD parameters and severity of MLS were assessed using Spearman's correlation coefficients. A two-sided *P* < 0.05 was considered statistically significant.

## Results

### Patients' Demographic and Clinical Characteristics

**Figure 2:**
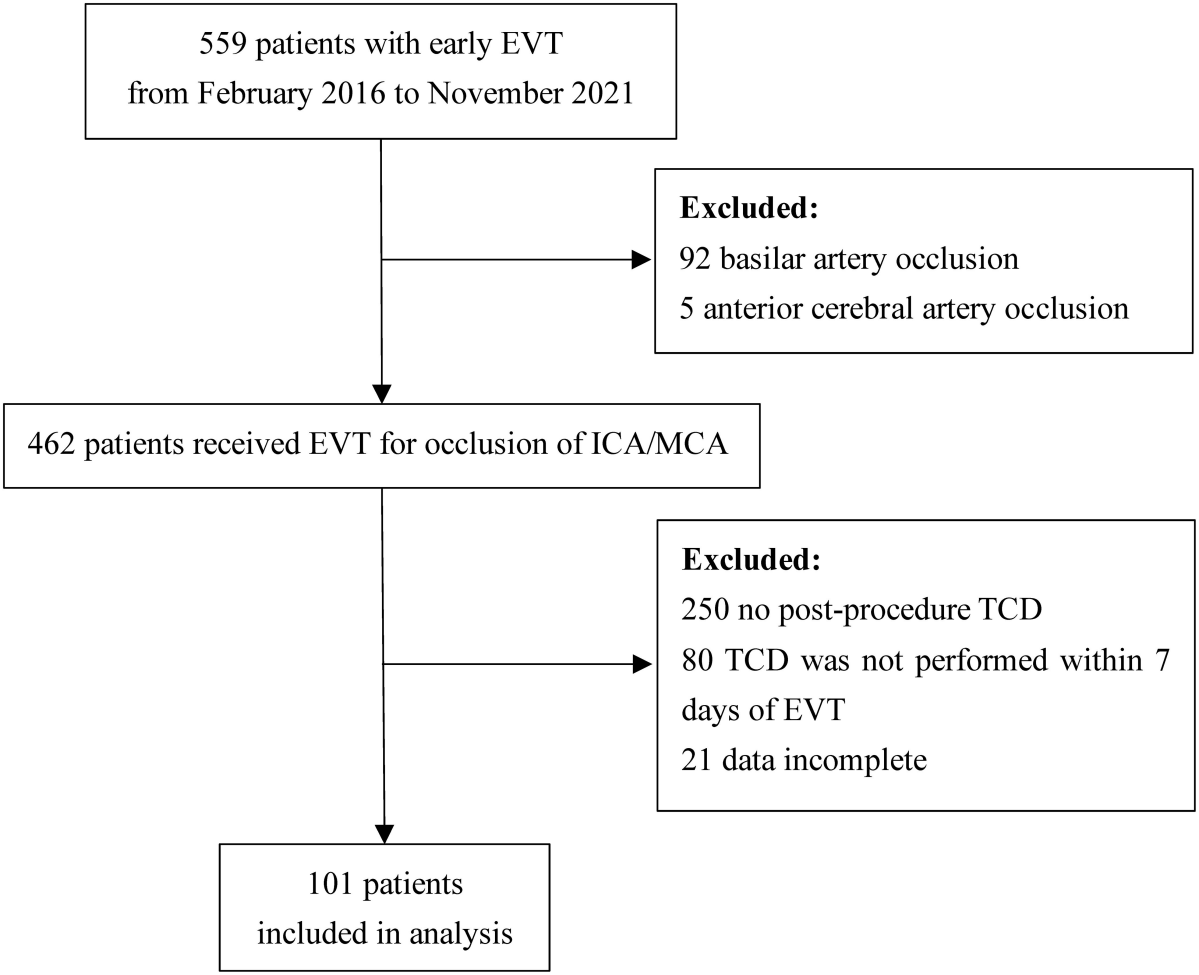
Flow diagram of patient inclusion for the study. EVT, endovascular therapy; ICA, internal carotid artery; MCA, middle cerebral artery; TCD, transcranial Doppler.

Details of patient inclusion are shown in Figure 2. Five hundred and fifty-nine patients received early EVT from February 2016 to November 2021 in our center. We excluded 92 patients for basilar artery occlusion, 5 patients for anterior cerebral artery occlusion, 250 patients for no post-procedure TCD, 80 patients for no TCD examination within 1 week of EVT, and 21 patients for incomplete data. Finally, a total of 101 patients were included in the analysis; 65.3% (66/101) were male and the mean age was 64.2 years.

The occluded artery of the index stroke was the ICA in 31.7% (32/101) of patients, the M1 segment of MCA in 47.5% (48/101), and the M2 segment of MCA in 20.8% (21/101). The median time from onset to TCD examination was 5 days (IQR: 3–6 days). Median systolic CBFV was 87 cm/s, median diastolic CBFV was 32 cm/s, and mean CBFV of the MCA on the affected side was 49.7 cm/s. The hemorrhagic transformation occurred in 42.6% (43/101) of patients within seven days of onset. Hemorrhagic infarction grade 1 affected 4% (4/101); hemorrhagic infarction grade 2, 19.8% (20/101); parenchymal hemorrhage grade 1, 14.9% (15/101); and parenchymal hemorrhage grade 2, 4% (4/101). Other baseline characteristics are shown in Table 1.

**Table 1:** Baseline characteristics and outcomes of included patients.

**Characteristic**	**Value**
Age, mean ± SD	64.2 ± 15.0
Male, *n* (%)	66 (65.3)
Hypertension, *n* (%)	62 (61.4)
Diabetes mellitus, *n* (%)	17 (16.8)
Atrial fibrillation, *n* (%)	49 (48.5)
Coronary heart disease, *n* (%)	7 (6.9)
Stroke history, *n* (%)	13 (12.9)
NIHSS on admission, median (IQR)	16 (13–20)
GCS on admission, median (IQR)	13 (12–14)
Duration from onset to TCD, days, median (IQR)	5.0 (3.0–6.0)
Intravenous thrombolysis, *n* (%)	42 (41.6)
Etiology, *n* (%)	
Large-artery atherosclerosis	31 (30.7)
Cardioembolism	55 (54.5)
Others	8 (7.9%)
Undetermined	7 (6.9)
Left hemisphere affected, *n* (%)	53 (52.5)
Occlusion site, *n* (%)	
ICA	32 (31.7)
M1	48 (47.5)
M2	21 (20.8)
Postoperative mTICI, *n* (%)	
<2b	3 (3.0)
2b	25 (24.8)
3	73 (72.3)
Affected MCA	
Systolic CBFV, cm/s, median (IQR)	87.0 (62.0–114.5)
Diastolic CBFV, cm/s, median (IQR)	32.0 (22.0–44.4)
Mean CBFV, cm/s, median (IQR)	49.7 (35.5–67.3)
PI, mean ± SD	1.1 ± 0.2
RI, mean ± SD	0.6 ± 0.1
Contralateral MCA	
Systolic CBFV, cm/s, median (IQR)	79.0 (67.5–97.5)
Diastolic CBFV, cm/s, median (IQR)	31.0 (23.0–38.1)
Systolic CBFV, cm/s, median (IQR)	47.0 (37.7–58.3)
PI, mean ± SD	1.1 ± 0.2
RI, mean ± SD	0.6 ± 0.1
Systolic CBFV ratio, median (IQR)	1.1 (0.9–1.4)
Diastolic CBFV ratio, median (IQR)	1.1 (0.8–1.5)
Mean CBFV ratio, median (IQR)	1.1 (0.8–1.4)
PI ratio, median (IQR)	1.0 (0.8–1.1)
RI ratio, median (IQR)	1.0 (0.9–1.1)
Malignant brain edema, *n* (%)	6 (5.9)
Severity of brain edema, *n* (%)	
No edema	44 (43.6)
CED-1	23 (22.8)
CED-2	10 (9.9)
CED-3	24 (23.8)

*All ratios indicate the proportion between affected/contralateral hemispheres*.

*CBFV, cerebral blood flow velocity; CED, cerebral edema; GCS, Glasgow Coma Scale; ICA, internal carotid artery; IQR, interquartile range; M1, horizontal segment of middle cerebral artery; M2, insular segment of middle cerebral artery; MCA, middle cerebral artery; mTICI, modified Treatment in Cerebral Ischemia; NIHSS, National Institutes of Health Stroke Scale; PI, pulsatility index; RI, resistance index; SD, standard deviation; TCD, transcranial Doppler*.

Brain edema was present on follow-up imaging in 56.3% (57/101) of included patients. CED-1 was present in 23 (22.8%) of included patients; CED-2, 10 (9.9%); and CED-3, 24 (23.8%). For patients with CED-3, the median value of MLS was 4 mm (IQR: 3–7 mm). Six patients developed MBE within seven days of stroke onset.

### Comparison of CBFV Between Patients Who Are Non-SBE and Patients With SBE

Compared to patients who are non-SBE (CED 0–1), patients with SBE (CED 2–3) had lower ratio of systolic CBFV (median: 1 vs. 1.2, *P* = 0.020), and lower ratio of mean CBFV (median: 0.9 vs. 1.3, *P* = 0.029). Similar trends were observed for the ratio of diastolic CBFV, but the intergroup difference did not reach statistical significance. Other baseline characteristics were comparable between the two groups (Table 2).

**Table 2:** Comparison of baseline characteristics between patients with or without severe brain edema (SBE).

**Characteristic**	**SBE** **(*n* = 34)**	**Non-SBE** **(*n* = 67)**	***P***
Age, mean ± SD	65.0 ± 14.4	63.9 ± 15.4	0.722
Male, *n* (%)	20 (58.8)	46 (68.7)	0.326
Hypertension, *n* (%)	21 (61.8)	41 (61.2)	0.956
Diabetes mellitus, *n* (%)	6 (17.6)	11 (16.4)	0.876
Atrial fibrillation, *n* (%)	20 (58.8)	29 (43.3)	0.140
Coronary heart disease, *n* (%)	1 (2.9)	6 (9.0)	0.418
Stroke history, *n* (%)	6 (17.6)	7 (10.4)	0.353
NIHSS at admission, median (IQR)	17 (15–19)	15 (12–20)	**0.048**
GCS at admission, median (IQR)	13 (12–14)	13 (12–14)	0.527
Duration from onset to TCD, days, median (IQR)	5 (3–6)	5 (3–6)	0.870
Intravenous thrombolysis, *n* (%)	15 (44.1)	27 (40.3)	0.713
Etiology, *n* (%)			0.529
Large-artery atherosclerosis	10 (29.4)	21 (31.3)	
Cardioembolism	21 (61.8)	34 (50.7)	
Others	1 (2.9)	7 (10.4)	
Undetermined	2 (5.9)	5 (7.5)	
Left hemisphere affected, *n* (%)	16 (47.1)	37 (55.2)	0.437
Occlusion site, *n* (%)			0.725
ICA	10 (29.4)	22 (32.8)	
M1	18 (52.9)	30 (44.8)	
M2	6 (17.6)	15 (22.4)	
Postoperative mTICI, *n* (%)			0.740
<2b	1 (2.9)	2 (3.0)	
2b	10 (29.4)	15 (22.4)	
3	23 (67.6)	50 (74.6)	
Systolic CBFV of affected MCA, cm/s, median (IQR)	84.0 (60.0–104.5)	88.0 (62.0–123.0)	0.327
Diastolic CBFV of affected MCA, cm/s, median (IQR)	31.5 (20.8–38.8)	33.0 (24.0–47.0)	0.394
Mean CBFV of affected MCA, cm/s, median (IQR)	47.7 (34.3–60.6)	51.0 (36.7–72.3)	0.304
PI of affected MCA, median (IQR)	1.0 (1.0–1.2)	1.0 (0.9–1.2)	0.752
RI of affected MCA, median (IQR)	0.6 (0.6–0.7)	0.6 (0.6–0.7)	0.752
Systolic CBFV of contralateral MCA, cm/s, median (IQR)	85.0 (71.5–107.3)	78.0 (66.0–94.0)	0.197
Diastolic CBFV of contralateral MCA, cm/s, median (IQR)	31.5 (22.0–42.0)	31.0 (23.0–38.0)	0.479
Mean CBFV of contralateral MCA, cm/s, median (IQR)	49.1 (41.3–62.3)	46.7 (37.3–55.7)	0.273
PI of contralateral MCA, median (IQR)	1.1 (0.9–1.3)	1.0 (0.9–1.2)	0.782
RI of contralateral MCA, median (IQR)	0.6 (0.6–0.7)	0.6 (0.6–0.7)	0.782
Systolic CBFV ratio, median (IQR)	1.0 (0.8–1.3)	1.2 (0.9–1.4)	**0.020**
Diastolic CBFV ratio, median (IQR)	1.0 (0.8–1.3)	1.3 (0.9–1.6)	0.050
Mean CBFV ratio, median (IQR)	0.9 (0.8–1.3)	1.3 (0.9–1.5)	**0.029**
PI ratio, median (IQR)	1.0 (0.9–1.1)	1.0 (0.8–1.2)	0.994
RI ratio, median (IQR)	1.0 (0.9–1.1)	1.0 (0.9–1.1)	0.903

*All ratios indicate the proportion between affected/contralateral hemisphere*.

*CBFV, cerebral blood flow velocity; GCS, Glasgow Coma Scale; ICA, internal carotid artery; IQR, interquartile range; M1, horizontal segment of middle cerebral artery; M2, insular segment of middle cerebral artery; MCA, middle cerebral artery; mTICI, modified Treatment in Cerebral Ischemia; NIHSS, National Institutes of Health Stroke Scale; PI, pulsatility index; RI, resistance index; SBE, severe brain edema; SD, standard deviation; TCD, transcranial Doppler. P-values in bold indicate statistically significant*.

After adjusting for potential confounders, including age, admission NIHSS score, and atrial fibrillation, we found increased risk of SBE to be independently associated with lower ratio of systolic CBFV (OR = 0.289, 95% CI: 0.069–0.861, *P* = 0.028) and lower ratio of mean CBFV (OR = 0.278, 95% CI: 0.084–0.914, *P* = 0.035) (Table 3). Among patients with MLS (CED-3), severity of MLS correlated with CBFV (systolic: *r* = −0.467, *P* = 0.021; diastolic: *r* = −0.465, *P* = 0.022; mean:

**Table 3:** Multivariate analysis of the association between the ratio of cerebral blood flow velocity and severe brain edema.

**Multivariate regression model**	**OR**	**95% CI**	***P***
**Model 1**			
Age	0.994	0.962–1.027	0.733
NIHSS score	1.083	0.993–1.181	0.072
Atrial fibrillation	1.709	0.666–4.385	0.265
Systolic CBFV ratio	0.289	0.069–0.861	**0.028**
**Model 2**			
Age	0.994	0.962–1.026	0.700
NIHSS score	1.078	0.990–1.173	0.084
Atrial fibrillation	1.677	0.657–4.279	0.280
Diastolic CBFV ratio	0.428	0.161–1.139	0.089
**Model 3**			
Age	0.994	0.962–1.027	0.717
NIHSS score	1.082	0.993–1.180	0.073
Atrial fibrillation	1.667	0.651–4.271	0.287
Mean CBFV ratio	0.278	0.084–0.914	**0.035**

*All ratios indicate the proportion between affected/contralateral hemispheres*.

*P-values in bold indicate statistically significant*.

*CBFV, cerebral blood flow velocity; CI, confidence interval; NIHSS, National Institutes of Health Stroke Scale; OR, odds ratio*.

**Figure 3:**
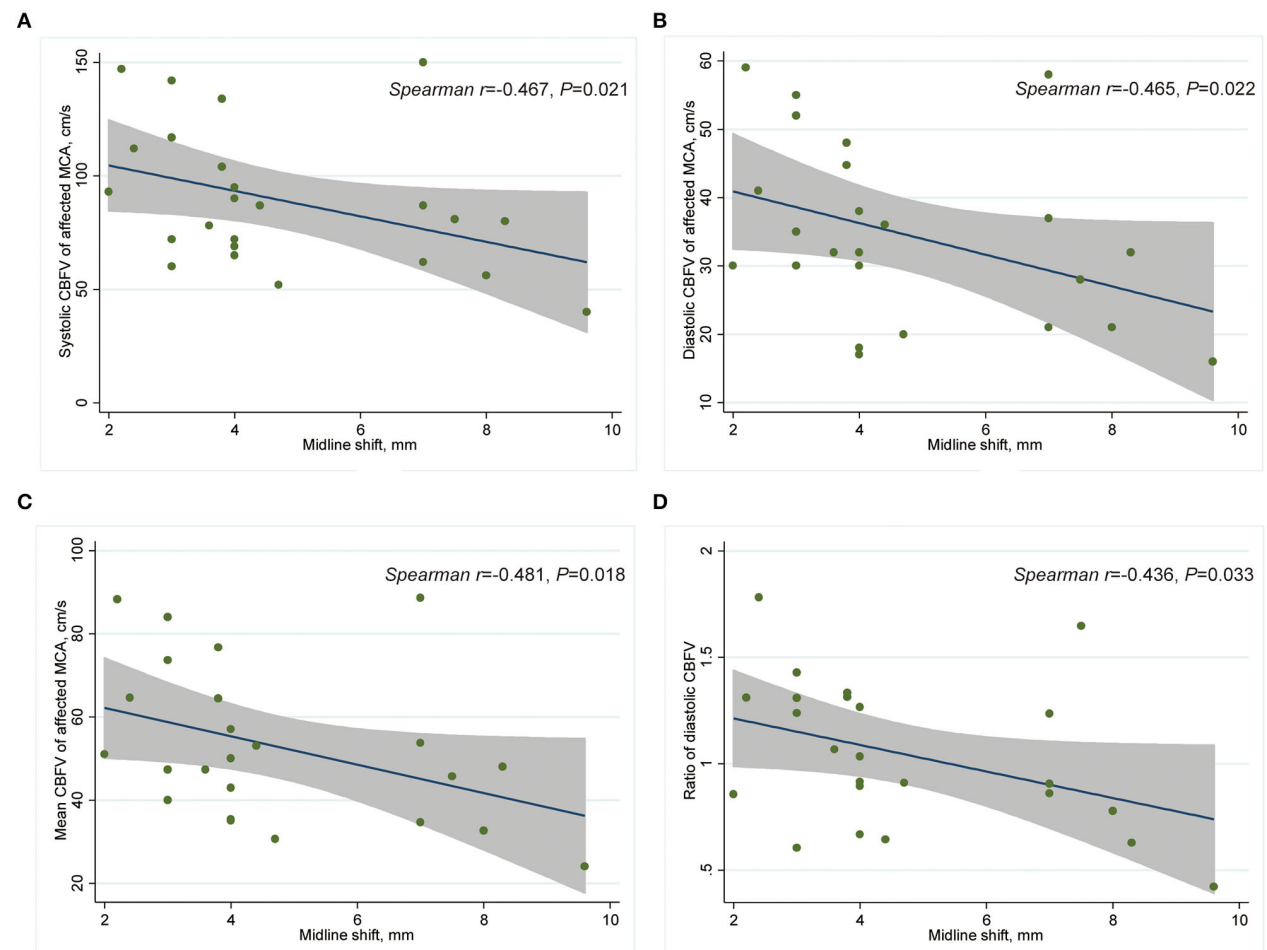
Scatter plot showing correlation between CBFV and MLS in patients with CED-3 (*n* = 24). **(A)** Systolic CBFV of the affected MCA and MLS; **(B)** diastolic CBFV of the affected MCA and MLS; **(C)** mean CBFV of the affected MCA and MLS; **(D)** and affected/contralateral ratio of diastolic CBFV and MLS. CBFV, cerebral blood flow velocity; CED, cerebral edema; MCA, middle cerebral artery; MLS, midline shift.

*r* = −0.481, *P* = 0.018) in the affected MCA and ratio of diastolic CBFV (*r* = −0.436, *P* = 0.033) (Table 4; Figure 3).

**Table 4:** Correlation between transcranial Doppler parameters and severity of midline shift in patients with CED-3 (*n* = 24).

**Transcranial Doppler parameter**	***r***	***P***
Affected MCA		
Systolic CBFV	−0.467	**0.021**
Diastolic CBFV	−0.465	**0.022**
Mean CBFV	−0.481	**0.018**
PI	0.164	0.444
RI	0.164	0.444
Contralateral MCA		
Systolic CBFV	−0.206	0.334
Diastolic CBFV	−0.075	0.728
Mean CBFV	−0.141	0.512
PI	−0.142	0.507
RI	−0.142	0.507
Systolic CBFV ratio	−0.300	0.155
Diastolic CBFV ratio	−0.436	**0.033**
Mean CBFV ratio	−0.372	0.073
PI ratio	0.255	0.229
RI ratio	0.259	0.223

*All ratios indicate the proportion between affected/contralateral hemispheres*.

*P-values in bold indicate statistically significant*.

*CBFV, cerebral blood flow velocity; CED, cerebral edema; MCA, middle cerebral artery; PI, pulsatility index; RI, resistance index*.

### Comparison of CBFV Between Patients Who Are Non-MBE and Patients With MBE

Compared to patients with non-MBE, patients with MBE had a lower ratio of systolic CBFV (0.8 vs. 1.1, *P* = 0.049) and a lower ratio of mean CBFV (0.8 vs. 1.1, *P* = 0.041) (Table 5). The multivariate analysis could not be performed due to the small number of patients in the MBE group.

**Table 5:** Comparison of baseline characteristics between patients with or without malignant brain edema (MBE).

**Characteristic**	**MBE** **(*n* = 6)**	**Non-MBE** **(*n* = 95)**	***P***
Age, median (IQR)	80.5 (59.8–83.3)	65.0 (55.0–74.0)	0.131
Male, *n* (%)	4 (66.7)	62 (65.3)	>0.999
Hypertension, *n* (%)	4 (66.7)	58 (61.1)	>0.999
Diabetes mellitus, *n* (%)	3 (50.0)	14 (14.7)	0.058
Atrial fibrillation, *n* (%)	6 (100.0)	43 (45.3)	**0.011**
Coronary heart disease, *n* (%)	0 (0)	7 (7.4)	>0.999
Stroke history, *n* (%)	1 (16.7)	12 (12.6)	0.572
NIHSS at admission, median (IQR)	18 (17–22)	16 (12–20)	0.091
GCS at admission, median (IQR)	13 (10–13)	13 (12–14)	0.167
Duration from onset to TCD, days, median (IQR)	3 (2–5)	5 (3–6)	0.142
Intravenous thrombolysis, *n* (%)	3 (50.0)	39 (41.1)	0.691
Etiology, *n* (%)			0.149
Large-artery atherosclerosis	0 (0)	31 (32.6)	
Cardioembolism	6 (100.0)	49 (51.6)	
Others	0 (0)	8 (8.4)	
Undetermined	0 (0)	7 (7.4)	
Left hemisphere affected, *n* (%)	3 (50.0)	50 (52.6)	>0.999
Occlusion site, *n* (%)			0.684
ICA	2 (33.3)	30 (31.6)	
M1	2 (33.3)	46 (48.4)	
M2	2 (33.3)	19 (20.0)	
Postoperative mTICI, *n* (%)			0.097
<2b	1 (16.7)	2 (2.1)	
2b	2 (33.3)	23 (24.2)	
3	3 (50.0)	70 (73.7)	
Systolic CBFV of affected MCA, cm/s, median (IQR)	80.5 (52.0–102.8)	88.0 (62.0–117.0)	0.417
Diastolic CBFV of affected MCA, cm/s, median (IQR)	30.0 (19.8–41.5)	32.0 (23.0–44.7)	0.610
Mean CBFV of affected MCA, cm/s, median (IQR)	46.8 (30.5–61.9)	50.0 (35.7–68.3)	0.490
PI of affected MCA, median (IQR)	1.0 (1.0–1.1)	1.0 (0.9–1.2)	0.966
RI of affected MCA, median (IQR)	0.6 (0.6–0.6)	0.6 (0.6–0.7)	0.966
Systolic CBFV of contralateral MCA, cm/s, median (IQR)	104.0 (61.3–136.3)	79.0 (68.0–96.0)	0.214
Diastolic CBFV of contralateral MCA, cm/s, median (IQR)	44.5 (24.5–58.0)	31.0 (23.0–38.0)	0.117
Mean CBFV of contralateral MCA, cm/s, median (IQR)	66.0 (36.3–81.6)	46.7 (37.7–56.3)	0.145
PI of contralateral MCA, median (IQR)	1.0 (0.8–1.2)	1.1 (0.9–1.2)	0.429
RI of contralateral MCA, median (IQR)	0.6 (0.5–0.7)	0.6 (0.6–0.7)	0.429
Systolic CBFV ratio, median (IQR)	0.8 (0.6–1.0)	1.1 (0.9–1.4)	**0.049**
Diastolic CBFV ratio, median (IQR)	0.7 (0.6–1.1)	1.2 (0.9–1.5)	0.056
Mean CBFV ratio, median (IQR)	0.8 (0.6–1.0)	1.1 (0.9–1.4)	**0.041**
PI ratio, median (IQR)	1.0 (0.9–1.3)	1.0 (0.8–1.1)	0.566
RI ratio, median (IQR)	1.0 (0.9–1.2)	1.0 (0.9–1.1)	0.481

*All ratios indicate the proportion between affected/contralateral hemispheres*.

*P-values in bold indicate statistically significant*.

*CBFV, cerebral blood flow velocity; GCS, Glasgow Coma Scale; ICA, internal carotid artery; IQR, interquartile range; M1, horizontal segment of middle cerebral artery; M2, insular segment of middle cerebral artery; MBE, malignant brain edema; MCA, middle cerebral artery; mTICI, modified Treatment in Cerebral Ischemia; NIHSS, National Institutes of Health Stroke Scale; PI, pulsatility index; RI, resistance index; TCD, transcranial Doppler*.

### Comparison of CBFV Between Hemorrhagic Transformation Subgroups

Compared to patients with hemorrhagic infarction, patients with parenchymal hemorrhage had a higher ratio of diastolic CBFV (1.3 vs. 0.9, *P* = 0.043), while values of CBFV were similar between patients with or without hemorrhagic transformation.

## Discussion

Patients with AIS with large vessel occlusion are at high risk of developing MBE even after EVT. However, reliable markers for the prediction of post-EVT brain edema severity are lacking. Thus, here, we aimed to investigate the association between post-procedure CBFV measured by TCD and the severity of brain edema to evaluate the prognostic capacity of CBFV. In a Chinese cohort of patients, we found that a lower ratio of affected/contralateral CBFV was independently associated with an increased risk of severe brain edema.

In AIS research, TCD has traditionally been used as a real-time screening tool for recanalization, perfusion state, and microemboli during EVT (22, 23), but studies on the association between TCD parameters and brain edema are limited. One study (24) assessed cerebral autoregulation using CBFV and arterial blood pressure in 46 patients with MCA infarction and found that impaired cerebral autoregulation was associated with brain edema on admission and 24 h after admission. In contrast, in this study, we assessed direct TCD parameters, which are more widely used in clinical practice than analysis of cerebral autoregulation.

In our study, a lower ratio of CBFV in the affected MCA relative to the contralateral MCA was associated with a higher risk of SBE. On the contrary, one study (25) included 185 patients with large vessel occlusion who received EVT, and found that a pathologically high ratio of peak systolic velocity (in the recanalized/contralateral MCA) was associated with 3-month poor outcomes, although the incidence of brain edema was not reported. Notably, the mean peak systolic velocity ratio was highest immediately after EVT and decreased with time in this study (25).

The detrimental effects of high CBFV could be explained by secondary injury due to cerebral hyperperfusion syndrome (26), while hypoperfusion itself is a strong risk factor for ischemia damage (27). Consistent with this, a study on traumatic brain injury showed that deviation of optimal cerebral perfusion pressure in either direction was associated with poor outcomes (28). Besides, contradictory results were observed when CBFV was measured at different times after EVT, suggesting that differences in when TCD was evaluated may be another reason for the observed disparity among studies. In a study of 123 patients with AIS receiving early EVT (29), the elevation of mean blood flow index (recanalized/contralateral MCA) within 24 h of EVT (mean time: 6.6 h) was associated with a higher risk of hemorrhagic transformation and unfavorable outcomes. While another study of 31 patients with AIS receiving EVT (30) reported that acceleration of CBFV within seven days (mean: 3.4 days) of mechanical thrombectomy was not associated with clinical deterioration.

Among TCD parameters, pulsatility index (PI) has been correlated with intracranial pressure in patients with traumatic brain injury (31). One study (32) investigated the association between PI generated from the contralateral MCA and MLS in patients with large MCA infarction. Researchers found that baseline PI values and their increase correlated with MLS on day 3, suggesting that higher PI was an indicator of increased intracranial pressure in AIS (32). In our study, however, PI values were similar between the SBE and non-SBE groups, which indicated that the relative decrease of CBFV in the affected hemisphere was not caused by the edema itself. We speculate that ischemia and mechanical injury to the vascular endothelium may cause dysfunction of cerebral autoregulation, which further aggravates ischemic damage and leads to brain edema (24, 33, 34).

Notably, although 33.7% of included patients in our study had SBE, only six of them developed MBE, which has a more profound influence on clinical outcomes (2). In our previous systematic review, the median time for detection of MBE in existing studies was 6 days (4). Here, the median time from onset to MBE was 5 days (2.8–5.3 days) and the median time from onset to TCD was 2.5 days (1.5–4.8 days) for patients with MBE. In an early post-mortem study (35), the extent of MLS reached its peak about 4 days after onset. A sonographic study detected a gradual increase in MLS during the first 4 days after hemispheric infarction (36). Therefore, CBFV measured by TCD early after EVT may offer valuable information for predicting space-occupying brain edema and subsequent MBE. However, this hypothesis could not be confirmed in the current study due to the small number of MBE cases.

Our study presents several limitations. First, the retrospective design might cause a selection bias, which may mean that some patients with severe edema did not undergo TCD because of their unstable condition. Second, the timing of TCD evaluations was not standardized. On the other hand, these two limitations may mean that our results better reflect actual clinical practice. Measuring CBFV immediately after EVT, and then, dynamically thereafter could help illuminate the natural course of brain edema following EVT and offer more information for clinical decisions. Prospective, multi-center studies with larger samples and quantitative analysis of brain edema are needed (37). A nomogram or grading scale derived from multivariate models is also needed to evaluate the reliability and strength of CBFV in predicting brain edema.

## Conclusion

A lower affected/contralateral ratio of CBFV in MCA within seven days of EVT may be associated with an increased risk of severe brain edema in patients with AIS. Post-procedure CBFV measured by TCD may be a predictor of severe brain edema.

## Data Availability Statement

The raw data supporting the conclusions of this article will be made available by the authors, without undue reservation.

## Ethics Statement

The studies involving human participants were reviewed and approved by Medical Ethics Committee of Zhejiang Provincial People's Hospital. The patients/participants provided their written informed consent to participate in this study.

## Author Contributions

HW, JP, and RY carried out the studies, participated in collecting data, and drafted the manuscript. YG and RY performed the statistical analysis and participated in its design. TW participated in the acquisition, analysis, or interpretation of data and drafted the manuscript. All authors read and approved the final manuscript.

## Funding

This work was supported by Projects of Medical and Health Science and Technology in Zhejiang Province of China (2019RC099, 2019KY010, 2021KY509, and 2022KY573) and the Scientific Research Fund of Zhejiang Provincial Education Department (Y201942650).

## Conflict of Interest

The authors declare that the research was conducted in the absence of any commercial or financial relationships that could be construed as a potential conflict of interest.

## Publisher's Note

All claims expressed in this article are solely those of the authors and do not necessarily represent those of their affiliated organizations, or those of the publisher, the editors and the reviewers. Any product that may be evaluated in this article, or claim that may be made by its manufacturer, is not guaranteed or endorsed by the publisher.
